# Comparison of two commercial broad-range PCR and sequencing assays for identification of bacteria in culture-negative clinical samples

**DOI:** 10.1186/s12879-017-2333-9

**Published:** 2017-03-27

**Authors:** Camilla Stavnsbjerg, Niels Frimodt-Møller, Claus Moser, Thomas Bjarnsholt

**Affiliations:** 1 Department of Clinical Microbiology, Centre for Diagnostics, Rigshospitalet, Copenhagen, Denmark; 2 Department of Immunology and Microbiology, Faculty of Health and Medical Sciences, University of Copenhagen, Copenhagen, Denmark

**Keywords:** Culture-negative samples, Molecular diagnostics, Universal Microbe Detection, 16S PCR

## Abstract

**Background:**

Culturing has long been the gold standard for detecting aetiologic agents in bacterial infections. In some cases, however, culturing fails to detect the infection. To further investigate culture-negative samples, amplification and subsequent sequencing of the 16S rRNA gene is often applied. The aim of the present study was to compare the current method used at our Department of Clinical Microbiology, based on the MicroSeq ID system (Applied Biosystems, USA) with the Universal Microbe Detection (UMD) SelectNA kit (Molzym, Germany).

**Methods:**

76 culture-negative samples were first processed with the MicroSeq ID analysis, where total DNA was extracted and the 16S gene amplified and sequenced with the MicroSeq ID system. Samples were subsequently processed with the UMD SelectNA analysis, where human DNA was removed during the DNA extraction procedure and the 16S gene amplified in a real-time PCR and sequenced.

**Results:**

22 of 76 samples (28.9%) were positive for bacteria with the UMD SelectNA, which was significantly more (*p* = 0.0055) than the MicroSeq ID where 11 of 76 samples (14.5%) were positive. The UMD SelectNA assay identified more relevant bacterial pathogens than the MicroSeq ID analysis (*p* = 0.0233), but also found a number of species that were considered contaminations.

**Conclusions:**

The UMD SelectNA assay was valuable for the identification of pathogens in culture-negative samples; however, due to the sensitive nature of the assay, extreme care is suggested in order to avoid false positives.

## Background

The standard method used in clinical microbiology laboratories for detection of bacterial and fungal pathogens is culturing of clinical specimens obtained from the patients. However, in some infectious episodes, the causative microorganism cannot be detected by culturing, even though the clinical signs and symptoms are suggestive of an infection. The occurrence of culture-negative results, when microorganisms are in fact present, can be due to prior antibiotic treatment [1], slow growing or fastidious microorganisms [2], or the presence of viable but nonculturable bacteria [3]. Culture-negative results are for instance seen with infective endocarditis [4], bone and joint infections including foreign body associated infections [5], otitis media [6], and meningitis [7].

Molecular methods have the potential to detect and identify pathogens that are nonculturable. One such method is PCR that identifies pathogens from their nucleic acids. Several types of PCRs can be used depending on purpose, such as species-specific PCRs, multiplex PCRs that targets a panel of species, or broad-range PCRs that potentially targets all species. At the Department of Clinical Microbiology at Rigshospitalet, part of a tertiary-referral hospital, samples are received from diverse infections with different etiologies. A broad-range PCR assay was implemented seven years ago in the department in order to identify pathogens in culture-negative samples from patients with a suspected infection. The method in use consists of a total genomic DNA extraction from the clinical samples followed by broad-range 16S rDNA PCR and sequencing analyses employing the MicroSeq ID kit. This led to an improved detection rate of infectious episodes when compared with culturing. However, a substantial number of samples still tested negative for microorganisms despite continued clinical suspicion of infection, indicating a suboptimal sensitivity.

The commercial broad-range UMD assay (also known as SepsiTest when performed on whole blood) has previously been shown to have an increased positivity rate compared to culturing for a variety of samples [8–11]. The assay includes a DNA extraction, in which human DNA is removed, followed by a PCR amplifying regions of the 16S and 18S genes. The assay has been reported by the company to have a lower detection limit of 20–460 colony forming units (CFU) per mL blood depending on species [12]. A semi-automated version of the assay, i.e. the UMD SelectNA, requiring less hands-on time has been developed since the previous studies. The aim of this study was to compare the MicroSeq ID analysis with the UMD SelectNA assay for the detection and identification of pathogens in culture-negative samples for routine diagnostic purposes.

## Methods

### Sample collection

Samples from patients with a suspected infection were collected under sterile conditions at either Rigshospitalet (a tertiary referral hospital, 1361 bed units, approximately 264,000 inpatients annually) or Hvidovre Hospital (a secondary referral hospital, 730 bed units, approximately 83,000 inpatients annually), and sent to the Department of Clinical Microbiology (DCM) at Rigshospitalet during the period of June 2015 to October 2015. Sample types included tissue adjacent to joints (27), other tissues (12), bone samples (9), body fluids (6), heart valves (5), joint fluids (5), foreign bodies (3), spinal fluids (3), pus (3), sonication fluids (2), E-swap (1). Culture-negative samples, from patients with a suspected infection, were subjected to MicroSeq ID 16S if requested by the clinical doctor responsible for the treatment of the patient. The same samples were subsequently processed with the UMD SelectNA assay. Specimens were stored at 5 °C upon arrival at the DCM, and processed within four weeks.

### Culturing

All growth media were obtained from Statens Serum Institute, Denmark. All samples were cultured for up to 5 days with the exception of heart valves that were cultured up to 14 days. For culture conditions see Table 1. The MALDI-TOF Biotyper (Bruker, Germany) was used to identify isolates.

**Table 1:** Culturing conditions

	Anaerobic plates (anaerobic conditions)	Chocolate agar plates (aerobic, 5% CO_2_)	Blood agar plates (aerobic, 5% CO_2_)	Eosin methylene blue plates (aerobic)	Brain heart infusion agar plates (anaerobic)	Thioglycolate broth (aerobic)	Serum bouillon broth (aerobic)
Soft tissue and body fluids	X	X	X	X		X	X
Bone samples	X	X	X	X		X	
Heart valves	X	X	X	X			
Pus samples		X	X	X	X	X	

### UMD SelectNA assay

#### DNA isolation

DNA isolation was performed with the UMD SelectNA CE-IVD kit (Molzym, Germany) according to the manufacturer’s recommendation. In short, tissue samples were pre-treated with proteinase K for 10 min. Body fluids, swabs and tissues were treated with a chaotropic buffer, lysing the human cells, and subjected to DNase treatment to degrade the human DNA. DNA from potentially present microorganisms was subsequently extracted in the SelectNA instrument and stored at −20 °C until real-time PCR analysis.

#### Real-time PCR

The real-time PCR assay was carried out with reagents from the UMD SelectNA kit. Two PCRs were performed for each sample: 1) a reaction amplifying the V3-V4 region of the bacterial 16S rDNA gene (481 bp); and 2) a control reaction amplifying a DNA fragment that was added during the DNA extraction to evaluate the efficiency of the extraction. The SYBR green based real-time PCR was perfomed in a LightCycler 480 instrument (Roche, Switzerland) with the following conditions: 95 °C for 1 min; 40 cycles of 95 °C for 5 s, 55 °C for 10s and 72 °C for 25 s followed by a melting curve analysis (70–95 °C). Samples with a melting temperature (T_m_) value between 87 and 91 °C for the 16S were considered positive.

#### Sequencing

PCR products from positive samples were purified with the QIAquick PCR purification kit (Qiagen, Netherlands). Sequencing primers included in the UMD SelectNA kit were used for Sanger sequencing, performed by Macrogen (Amsterdam, Netherland). Sequences were initially aligned to the SepsiTest BLAST database (http://www.sepsitest-blast.de) and subsequently if no bacterial species was identified by the database, to the NCBI BLAST database (http://www.ncbi.nlm.nih.gov/BLAST/). Sequences with ≥97–99% or ≥99% identity to the database were assigned to genus level or species level, respectively. Mixed bacterial sequences were analyzed with the RipSeq mixed program (Pathogenomix, USA) to resolve the individual sequences [13].

#### Precautions to avoid contamination

DNA isolation and addition of DNA to the PCR was carried out in a laminar flow bench in a room designated for extraction procedures. The master mix was prepared in a laminar flow bench in a DNA-free master-mix room. All surfaces of benches and equipment were cleaned with the disinfectant Virkon (Virkon Disinfectant Technologies, UK) before use. A 1 h UV decontamination program was performed on the SelectNA instrument before every use. Sterile elbow-long gloves and designated lab coats were used at all times.

### MicroSeq ID 16S analysis

#### DNA isolation

Samples were processed with the DNeasy Blood and Tissue kit (Qiagen, Netherlands) according to the manufacturer’s tissue protocol with a few modifications: after 56 °C incubation with proteinase K for 2–3 h, samples were incubated for additional 10 min at 95 °C, and the final elution volume was 100 μL AE buffer. DNA solutions from all tissue samples were diluted 1:40 in nuclease-free water.

#### PCR

The MicroSeq 500 16S rDNA PCR (Applied Biosystems, United States) was used to amplify the V1-V2 region of the 16S. Sample DNA and the master mix were mixed in a 1:1 ratio and the reaction was carried out in a 9800 Fast Thermal Cycler (Applied Biosystems, USA) with the following conditions: initial denaturation of 95 °C for 10 min and 30 cycles of 95 °C for 30s, 60 °C for 30 min and 72 °C for 45 s, followed by a final extension of 72 °C for 10 min. PCR products were resolved by electrophoresis on a 2% agarose gel (Embi Tech, United States). Samples with a 500–600 bp PCR fragment were purified with the ExoSap-IT kit (Affymetrix, United States).

#### Sequencing

PCR products were sequenced with the MicroSeq 500 16S Seq kit (Applied Biosystems, United States) according to manufacturer’s instructions. Products were then purified with gel filtration cartridges (Edge Bio, Unites States) and sequenced on a 3130xl Genetic Analyzer (Applied Biosystems, Unites States). The MicroSeq ID software was used to identify the species and in cases with low match % the sequence was additionally aligned to the NCBI BLAST database.

#### Precautions to prevent contamination

DNA extraction and PCR setup was performed in a pre-amplification room separated from the post-amplification room where the sequencing was performed. Gloves and designated lab coats were applied.

### Evaluation of results and statistics

A comparison was performed on the sequencing results obtained by the two methods and other findings from the same patient, including: culturing, molecular analyses, histopathology and serology. A finding was considered a relevant pathogen when the same organism was identified in another sample from the same patient by culturing or molecular analysis, or when histopathology or serology supported the finding. Findings with no such support were considered ambiguous. McNemar’s test was performed on 2 × 2 contingency Tables. A *p*-value below 0.05 was considered significant.

## Results

### Positivity rate of the two methods

76 culture-negative samples originating from 46 patients were processed with both the MicroSeq ID analysis and the UMD SelectNA assay. A total of 22 samples (28.9%) were positive with the UMD SelectNA assay, which was significant more compared to the 11 positive samples (14.5%) with the MicroSeq ID (*p* = 0.0055) (Table 2).

**Table 2:** Summary of all results

All samples	MicroSeq ID			
UMD SelectNA		+	−	Total
	+	10	12	22
	−	1	53	54
	Total	11	65	76

+ = positive, − = negative. A McNemar’s test was performed, *p* = 0.0055

### Concordance

Ten samples were positive with both the MicroSeq ID analysis and the UMD SelectNA analysis (Table 3). The same organisms were found with the two methods with the exception of one pus sample from a cerebral abscess, which showed only partial concordance. The MicroSeq ID identified *Fusobacterium nucleatum* and *Parvimonas micra* while the UMD SelectNA identified *Dialister pneumocitis* and *Parvimonas micra* (ID 3). All identified species are common pathogens found in abscesses with an odontogenic origin and therefore considered clinically relevant [14].

**Table 3:** Concordant result of the UMD SelectNA and MicroSeq ID methods

Patient ID	Sample type	Indication	UMD SelectNA result	MicroSeq ID result	Other findings^a^
1	Aorta tissue	Endocarditis	*Streptococcus pyogenes*	*S. pyogenes*	*S. pyogenes* cultured from blood sample
2	Heart valve tissue	Endocarditis	*Staphylococcus aureus/simiae*	*S. aureus*	*S. aureus* cultured from blood sample
3	Pus	Cerebral abscess	*Dialister pneumocitis* & *Parvimonas micra*	*Fusobacterium nucleatum* & *P. micra*	*F. nucleatum* cultured from blood sample
4	Seroma fluid	Breast cancer	*Streptococcus mitis/oralis*	*S. mitis*	
5	Pus	Abscess in fossa iliaca	*Streptococcus intermedius*	*S. intermedius*	
6	Tissue	Infected hip prosthesis	*Streptococcus dysgalactiae equisimilis*	*S. dysgalacticae equisimilis*	
	Tissue		*S. dysgalactiae equisimilis*	*S. dysgalacticae equisimilis*	
	Tissue		*S. dysgalactiae equisimilis*	*S. dysgalacticae equisimilis*	
7	Aorta valve	Endocarditis	*S. pyogenes*	*S. pyogenes*	
8	Aorta valve	Endocarditis	*Streptococcus anginosus*	*S. anginosus*	*S. anginosus* cultured from blood samples

^a^Findings from other sample taken from the same patient

### Discordance

Twelve samples were positive with the UMD SelectNA analysis and negative with the MicroSeq ID analysis, whereas only one sample was positive with the MicroSeq ID analysis and negative with the UMD SelectNA analysis (Table 4). The results were compared to other findings from the patients obtained within the last year in order to evaluate the relevance of the findings, and based on this categorized as either relevant findings (R) or ambiguous findings (A).

**Table 4:** Discordant result of the UMD SelectNA and MicroSeq ID methods

Patient ID	Sample type	Indication	UMD SelectNA result	MicroSeq ID result	Other findings	Conclusion
6	Tissue	Infected hip prosthesis	*S. dysgalactiae equisimilis*	−	*S. dysgalactiae equisimilis* found with 16S in 3 other samples from the patient	R
9	Tissue	Mycotic aneurism	*S. pneumoniae*	−	Cocci found in blood culture bottle and positive antibody reaction for pneumococci	R
10	Tissue	Spinal implant infection	*Staphylococcus lundunensis*/*hominis*	−	*Staphylococcus hominis* cultured from tissue sample	R
11	Tissue	Infected knee prosthesis	*S. aureus*/*simiae*	−	*S. aureus* cultured from tissue	R
	Tissue		*S. aureus*/*simiae*	−		R
12	Tissue	Infected hip prosthesis	*Staphylococcus epidermidis*/*caprae*/*capitis*	−	*S. epidermidis* cultured in 3 out of 5 biopsies	R
						R
	Tissue		*S. epidermidis*/*caprae*/*capitis*	−		
13	Tissue	Cerebral abscess	−	*Toxoplasma gondii* (parasite)		R
14	Tissue	Infected hip prosthesis	*Bacillus sp.* & *Thiotrix sp.*	−	Two other tissue samples negative with the UMD and routine 16S/28S	A
15	Fluid from pacemaker electrode	Pericarditis	*Kocuria rhizophila*	−	Two other samples negative with UMD and routine 16S/28S	A
16	Implantation material	Spondylodiscitis	*Hymenobacter arizonensis*	−	Other implant material sample negative with UMD and routine 16S/28S	A
17	Tissue	Spondylodiscitis	*Cloacibacterium normanense* & *Bergeyella sp. H1890*	−	Fluid from back negative with UMD and routine 16S/28S	A
18	Lymph node	Lymphoma	*S. epidermidis*	−		A

The UMD SelectNA identified relevant pathogens in 7 samples (ID 6, 9–12) of which the MicroSeq ID analysis were negative. One relevant pathogen, i.e. *Toxoplasma gondii*, was only detected by the MicroSeq ID (ID 13, cerebral abscess). The number of positive samples with relevant bacterial species were significantly higher using the UMD SelectNA compared to the MicroSeq ID (*p* = 0.0233) (Table 5). In the remaining 5 samples bacterial species were considered ambiguous findings because the results were not confirmed by other findings. The *Bacillus* species and *Thiothrix flexilis* detected in tissue located adjacent to an infected prosthesis (ID 14) were most likely contaminants as where the *Staphylococcus epidermidis* found in a lymph node from a lymphoma patient (ID 18). *Kocuria rhizophila* (ID 15, pericarditis) and *Bergeyella sp. H1890* (ID 17, spondylodiscitis) have been reported to cause human disease [15, 16] while *Cloacibacterium normanense* (ID 17, spondylodiscitis) and *Hymenobacter arizonensis* (ID 16, spondylodiscitis) are not typically associated with human infections, but have been detected at surgical wound sites [17].

**Table 5:** Results of samples with relevant bacterial species only

Samples with relevant bacteria	MicroSeq ID			
UMD SelectNA		+	−	Total
	+	10	7	17
	−	0	59	59
	Total	10	66	76

+ = positive for a relevant bacterial species, − = negative for a relevant bacterial species. A McNemar’s test was performed, McNemar’s test *p* = 0.0233

## Discussion

Seventy-six culture-negative samples from patients with suspected infection were analyzed with the MicroSeq ID analysis and the UMD SelectNA analysis. The UMD SelectNA method identified significantly more relevant bacterial pathogens than the MicroSeq ID analysis, and thus proved to be a more sensitive analysis for detecting bacteria. It is possible that the removal of human DNA during the UMD SelectNA method explains the higher sensitivity, since excessive amounts of human DNA has been shown to inhibit the 16S PCR reaction [18]. The singule case (ID 13) where the MicroSeq ID analyses detected a pathogen not detected by the UMD SelectNA analysis was in a pus sample from a cerebral abscess. In this case the intracellular parasite *Toxoplasma gondii* was identified as demonstrated earlier with this method [19]. It is possible that the chaotropic buffer intended to lyse the human cells during the UMD SelectNA DNA extraction disrupted the membrane of the parasite and exposed the DNA to the DNase activity.

In another case of cerebral abscess (ID 3) the two methods identified different oral-cavity-derived bacterial species that were all considered relevant. Often multiple species are associated with cerebral abscesses of odontogenic origin [14], and it is well known that Sanger sequencing of the 16S gene is limited to detecting two or perhaps three species [20]. In order to obtain a higher resolution, next generation sequencing must be applied.

In addition to the relevant pathogens, the UMD SelectNA method also identified a number of ambiguous findings. The broad-range nature of the assay combined with the low detection limit make the assay sensitive to contamination, since only a few bacteria introduced during sampling, handling or processing of the sample will give rise to a positive result. A limitation in this study was that the UMD SelectNA assay was performed on leftover material. Patient samples were primarily used for culture-based identification, subsequently for the 16S MicroSeq ID analyses and finally for the UMD SelectNA assay. Therefore, it is possible that samples were contaminated in the process of handling. However, similar studies evaluating the manual version of the UMD assay found a corresponding number of unlikely bacteria in different clinical specimens that they considered environmental contaminants [21, 22]. Haag et al. [21] states that material already processed for other purposes than molecular diagnosis is of limited value and that samples should be spilt upon arrival to the laboratory . However, at our Department of Clinical Microbiology, molecular analysis is often ordered after culturing has proven negative, and due to the large number of samples received every day it is not feasible to divide all the samples in case they are send for molecular analysis later. Thus, in this study we have demonstrated that samples used for culturing can still be of value for detecting relevant pathogens with molecular methods. Also, it cannot be excluded that the rare bacterial species identified with the UMD SelectNA assay are causative of the infection. Indeed, these events should be monitored to elucidate their true nature.

Samples were stored at 5 °C to avoid potential cell disruption from freezing and thawing that would result in digestion of exposed microbial DNA and the occurrence of false negatives with the UMD SelectNA. To evaluate the possible contamination risk of storing the samples at this temperature, seven bone samples from healthy donors where processed with the UMD SelectNA both upon arrival at the Department of Clinical Microbiology and 4 weeks later. All 16S real-time PCR reactions were negative, indicating that no contamination had occurred (data not shown).

Even with the increased sensitivity of the SelectNA analysis the majority of the samples were negative (70.7%). This could be due to 1) the patient did not have an infection, 2) the amount of bacteria in the sample was below the detection limit of the method or 3) the sample did not contain any bacteria even though the patient had an infection. Many of the samples in this study were collected from patients with chronic infections where it has been shown that the bacteria are not homogeneously distributed at the infectious site [23, 24], and that they can be caused by bacteria growing in small biofilms down to 5 μm [25]. In these cases, sampling of the infected area is extremely difficult. At least two patients with negative results did have an infection. Three bone samples from one patient, suspected of having a tuberculosis osteomyelitis, were positive with a *Mycobacterium-tuberculosis*-specific PCR performed at another diagnostic laboratory (State Serum Institute, SSI). Additionally, another patient suspected of having an atypical mycobacterial infection tested positive for *Mycobacterium avium* at the SSI. Mycobacteria species are included in the list of bacteria that can be detected by the assay, but the thick cell wall of mycobacteria species has previously proved to be difficult to disrupt [26, 27]. It is possible that the chemical and enzymatic lysis in the UMD SelectNA assay is not sufficient for disrupting the mycobacterial cells.

This study is, to the authors’ knowledge, the first to report on the performance of the semi-automated version of UMD assay, i.e. the SelectNA, which has a definite shorter hands-on time than the manual version. Comparison of this study to previously published studies of the manual version is somewhat difficult due to the difference of study design and sample types. All previous studies have compared the UMD assay (or SepsiTest) with culturing, in contrast to this study where all samples included were culture negative, consequently they were able to calculate sensitivity and specificity of the method by comparing to culturing results. Whether it is relevant to use culturing as the gold standard when culturing does not find all pathogens is debatable. Here it was chosen not to calculate sensitivity and specificity for the methods in lack of a reliable gold standard. Kühn et al. applied the method for diagnosis of infectious endocarditis and reported a sensitivity of 85% with the UMD [11]; this study likewise found a high positivity rate for heart valves with three out of five positive for relevant pathogens. Haag et al. found 37.9% culture-negative but PCR positive with the UMD assay, which is more than the 28.9% found in this study [21]. A possible explanation of this discrepancy might be that Rigshospitalet is a large tertiary referral hospital with patients suffering from rare diseases where the etiology is difficult to elucidate. In any case both studies demonstrate that the UMD is applicable to many different samples types, which is convenient in a diagnostic laboratory receiving many different sample types. Recently, a new version of the SelectNA instrument fully automating the DNA extraction for fluid samples and only requiring a short manual pre-treatment for tissue has been developed. The DNA extraction involves less hands-on time and is currently being tested at the Department.

## Conclusion

In summary, the UMD SelectNA assay was found to be more sensitive than the MicroSeq ID analysis currently used at the Department of Clinical Microbiology, and therefore valuable for detecting pathogens in culture-negative samples. Because of the increased sensitivity, the assay is sensitive to contamination. Therefore, aseptic handling of the samples and a thorough clinical evaluation of the patient’s history is vital in order to assess the relevance of the findings.

## Abbreviation

PCR: Polymerase chain reaction
UMD: Universal Microbe Detection
